# Bioorthogonal click chemistry for fluorescence imaging of choline phospholipids in plants

**DOI:** 10.1186/s13007-018-0299-2

**Published:** 2018-04-18

**Authors:** Janet M. Paper, Thiya Mukherjee, Kathrin Schrick

**Affiliations:** 1 Division of Biology, Kansas State University, Manhattan, KS 66506 USA; 2 Molecular, Cellular and Developmental Biology, Kansas State University, Manhattan, KS 66506 USA; 3 Department of Biochemistry and Molecular Biophysics, Kansas State University, Manhattan, KS 66506 USA; 4 Present Address: Department of Biology, Benedictine College, Atchison, KS 66002 USA

**Keywords:** Phosphatidylcholine, Propargylcholine, Phospholipids, Click chemistry, *Arabidopsis thaliana*, Fluorescence labeling, ESI-MS/MS

## Abstract

**Background:**

Phospholipids are important structural and signaling molecules in plant membranes. Some fluorescent dyes can stain general lipids of membranes, but labeling and visualization of specific lipid classes have yet to be developed for most components of the membrane. New techniques for visualizing membrane lipids are needed to further delineate their dynamic structural and signaling roles in plant cells. In this study we examined whether propargylcholine, a bioortholog of choline, can be used to label the major membrane lipid, phosphatidylcholine, and other choline phospholipids in plants. We established that propargylcholine is readily taken up by roots, and that its incorporation is not detrimental to plant growth. After plant tissue is harvested and fixed, a click-chemistry reaction covalently links the alkyne group of propargylcholine to a fluorescently-tagged azide, resulting in specific labeling of choline phospholipids.

**Results:**

Uptake of propargylcholine, followed by click chemistry with fluorescein or Alexa Fluor 594 azide was used to visualize choline phospholipids in cells of root, leaf, stem, silique and seed tissues from *Arabidopsis thaliana*. Co-localization with various subcellular markers indicated coinciding fluorescent signals in cell membranes, such as the tonoplast and the ER. Among different cell types in the leaf epidermis, guard cells displayed strong labeling. Mass spectrometry-based lipidomic analysis of the various plant tissues revealed that incorporation of propargylcholine was strongest in roots with approximately 50% of total choline phospholipids being labeled, but it was also incorporated in the other tissues including seeds. Phospholipid profiling confirmed that, in each tissue analyzed, incorporation of the bioortholog had little impact on the pool of choline plus choline-like phospholipids or other lipid species.

**Conclusion:**

We developed and validated a click-chemistry based method for fluorescence imaging of choline phospholipids using a bioortholog of choline, propargylcholine, in various cell-types and tissues from *Arabidopsis*. This click-chemistry method provides a direct way to metabolically tag and visualize specific lipid molecules in plant cells. This work paves the way for future studies addressing *in situ* localization of specific lipids in plants.

**Electronic supplementary material:**

The online version of this article (10.1186/s13007-018-0299-2) contains supplementary material, which is available to authorized users.

## Background

Phospholipids are major structural components of cell membranes. These lipids are not only critical for membrane integrity, as they can form lipid bilayers, but they also play specific roles in development and may serve as signaling molecules. In eukaryotic cells, phosphatidylcholine (PC) is generally considered to be the most abundant membrane phospholipid [1]. In plants, PC serves the above-mentioned functions as well as responds to abiotic stresses. PC increases when exposed to cold and *Arabidopsis* genes required for PC biosynthesis show elevated expression in response to both cold and salt [2, 3]. PC levels are also implicated in plant-specific developmental pathways. Concentrations of PC in the shoot apical meristem are correlated with flowering time and the *Arabidopsis* FLOWERING LOCUS T (FT), a florigen protein, was shown to bind PC [4].

The major site for phospholipid synthesis is the endoplasmic reticulum with phosphatidic acid (PA) as the common precursor [5]. In addition, PA is produced when the choline head group is cleaved by phospholipase. PA itself is implicated in numerous signaling pathways in plants, notably in mediating water and salt stress responses [6]. In the eukaryotes including plants, PC is synthesized via the CDP-choline pathway using diacylglycerol as the substrate and/or via the phosphatidylethanolamine (PE) methylation pathway [1, 7]. The CDP-choline pathway, which involves serial reactions catalyzed by choline kinase and other enzymes that act on choline, is the main route of synthesis in mammals [1]. The PE methylation pathway is the primary route of synthesis in yeast but plant PC synthesis is derived from both pathways [1, 7].

Although much is known about the enzymes that synthesize and metabolize phospholipids, there are limited ways of visualizing phospholipids within the membrane. Historically, lipids and membranes have been visualized using fluorescent dyes. Nile red is a general lipid staining dye that fluoresces in a hydrophobic but not in aqueous environments. It is utilized to pinpoint sites of lipid accumulation [7, 8]. FM4-64 or FM1-43 present examples of other dyes that when targeted to a lipid or when bound to membranes fluoresce strongly [7, 9]. While useful as counterstains for the plasma membrane and other lipid-rich structures, these dyes are general and not able to distinguish between the distinct types of membrane phospholipids. On the other hand, 1,2-bis(4,4-difluoro-5,7-dimethyl-4-bora-3a,4a-diaza-s-indacene-3-undecanoyl)-snglycero-3-phosphocholine (Bodipy PC), is a substrate leading to fluorescent undecanoic acid and a lysophosphatidylcholine which have been used as an *in vivo* endocytic marker [10], and as a marker for the ER and lipid droplets [11]. However, Bodipy probes cannot be used to replace existing lipid pools.

A novel method to incorporate and visualize choline phospholipids using the choline analog propargylcholine was previously established for mammalian cells and whole animals [12]. As a bioortholog, propargylcholine is biosynthetically incorporated into cell membranes and can be detected by cycloaddition with a fluorescent molecule attached to an azide group via a click-chemistry reaction [13, 14]. Applying this method, fluorescence microscopy was used to image choline phospholipids with propargylcholine incorporation in cell cultures and within organs of whole mice [12]. Lipidomic profiling demonstrated strong incorporation of the analog, which substitutes for choline at a rate of up to 44% when a concentration of 500 μM was used, while showing no significant impact on the ratios of non-choline lipids [12]. This method has been applied to visualize changes in choline uptake in assaying the toxicity of cisplatin to cancer cells and in comparing choline uptake between cancerous and non-cancerous biopsy samples [15, 16]. Propargylcholine has also been used to study the catabolism of choline phospholipids in the pathogen *Pseudomonas aeruginosa* [17].

In the present study we investigated the ability of the bioortholog propargylcholine to label plant choline phospholipids. Since this bioortholog is water soluble, it can easily be added to an agar medium. *Arabidopsis thaliana* seedlings grown on medium supplemented with propargylcholine were found to readily take up propargylcholine through the roots. Incorporation of the bioortholog had no adverse affects on plant growth and little or no alteration of other lipid species in various tissues. Using a click-chemistry methodology, we fluorescently labeled and visualized choline phospholipids in the root, stem, leaf, and seed. This method opens the door to more detailed studies that address the dynamic role of choline phospholipids, and potentially other lipids, in plant growth and development.

## Results

### Propargylcholine labels choline phospholipids in *Arabidopsis* seedlings

**Fig. 1:**
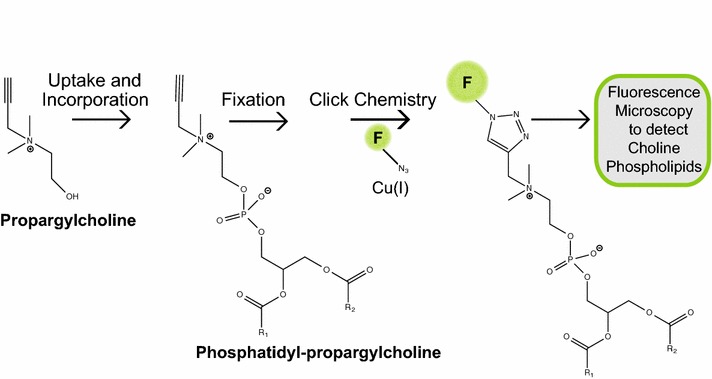
Schematic for bioorthoganal labeling and detection of choline phospholipids in plants. Steps are indicated by arrows, from left to right. In the first step, the propargylcholine label is taken up and biosynthetically incorporated into plant cell membranes to produce phosphatidyl-propargylcholine. In the second step, the plant material is harvested and fixed. The next step is to perform a copper(I)-catalyzed click-chemistry reaction in which a fluorophore (F) such as fluorescein (or Alexa Fluor 594) attached to an azide moiety is added to the terminal alkyne group on phosphatidyl-propargylcholine. In the final step, fluorescence microscopy is used to evaluate the tissue distribution and subcellular localization of choline phospholipids

We examined whether choline phospholipids from seedlings can be labeled with the choline bioortholog propargylcholine. We reasoned that propargylcholine can be taken up via the root system and biosynthetically incorporated into phosphatidylpropargylcholine. After growth on media containing the bioortholog, plants are harvested, fixed, and subjected to click chemistry with a fluorescently-tagged azide as illustrated in Fig. 1. We germinated *Arabidopsis* seeds on a defined media supplemented with (treated) and without (untreated control) propargylcholine and allowed seedlings to grow for 7 days (see “Methods”). A concentration of 250 μM propargylcholine was chosen for our study, based on the finding that 100–250 μM was sufficient for labeling in mammalian cells [12].

**Fig. 2:**
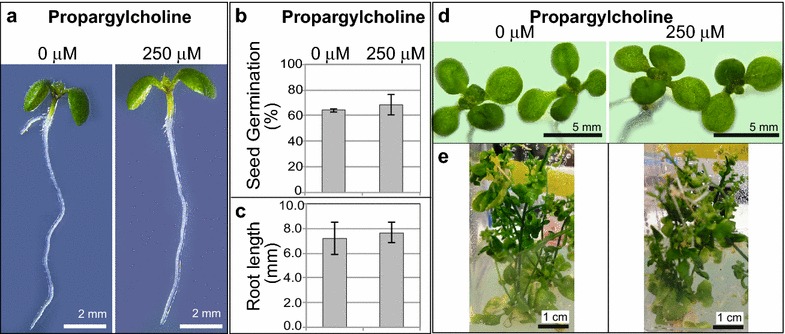
Propargylcholine labeling does not adversely affect plant growth. **a**–**e** *Arabidopsis* plants were germinated and grown on agar medium either with 0 µM propargylcholine (negative control), or with **a**–**d** 250 μM or **e** 200 μM propargylcholine. **a** Root lengths were compared for 7-day-old seedlings grown in the presence or absence of propargylcholine. **b** Percent seed germination was not significantly different on media containing propargylcholine versus the control. Error bars indicate the standard deviation (SD) for the mean of three replicates (n ≥ 27; two-tailed *t*-test, P = 0.38), **d** Growth on 250 μM propargylcholine did not affect root length. Error bars denote the SD for the mean of three replicates (n ≥ 9; two-tailed *t*-test, P = 0.64), **d** Fourteen-day-old seedlings grown on propargylcholine exhibited similar emergence of cotyledons and first leaves as the control. **e** Six-week-old plants grown in Magenta boxes display comparable growth physiology at the reproductive phase of development

Seedling growth was not adversely affected by growth in media containing 250 μM propargylcholine (Fig. 2a). Seed germination efficiency was also not affected by 250 μM propargylcholine (Fig. 2b). We measured root lengths for seedlings grown in the absence or presence of 250 μM propargylcholine, and did not detect a significant difference (Fig. 2c). There were no obvious growth or developmental differences between the treated and control seedlings at later stages (Fig. 2d, e).

**Fig. 3:**
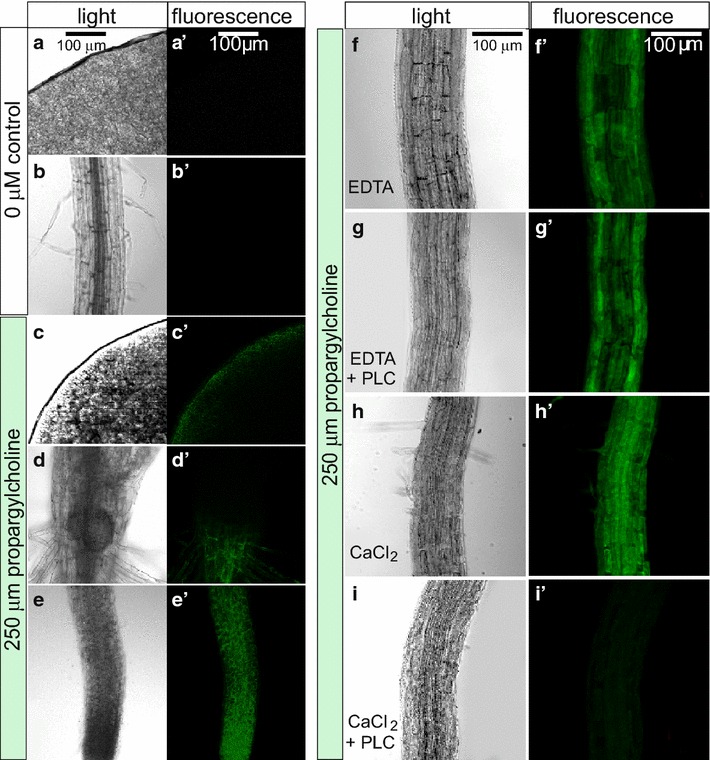
Visualization of labeled choline phospholipids in seedlings. *Arabidopsis* seeds were germinated and seedlings were grown for 7 days on agar media containing **a**, **b** 0 µM propargylcholine (negative control) or **c**–**i** 250 µM propargylcholine. **a**–**i** Light microscopy shows morphology of the tissues analyzed. **a**′–**i**′ Fluorescence signals are indicated in green. **a** Untreated cotyledon and **b** untreated root exhibit no visible background fluorescence. **c**–**f** Fluorescence marks the presence of propargylcholine in **c** cotyledons, **d** hypocotyl-root junction, and **e** root. **f**–**i** Propargylcholine labeling is removed by Phospholipase C treatment. *Arabidopsis* seedlings grown on 250 µM propargylcholine were prepared for click chemistry with fluorescein azide followed by treatment of roots with **f** EDTA alone, **g** EDTA with a bacterial phospholipase C (PLC), **h** CaCl_2_ alone, and **i** CaCl_2_ with PLC. The PLC enzyme removes choline head groups from phospholipids and requires calcium for activity. **i** In the presence of PLC and calcium, the fluorescein azide labeling of propargylcholine is markedly reduced. Scale bars shown in **a** and **f** are constant for **a**–**e** and **f**–**i**, respectively

Seedlings were fixed and subjected to a click-chemistry reaction to attach fluorescein azide to labeled choline phospholipids (Fig. 1; see “Methods” section). Fluorescence signals in seedling tissues were visualized by confocal laser scanning microscopy (Fig. 3). Control seedlings grown in the absence of propargylcholine lacked fluorescein signals in the cotyledons or roots when monitored at the same microscope settings (Fig. 3a, b). Seedlings that were grown in the presence of 250 μM propargylcholine exhibited fluorescence in all tissues examined (Fig. 3c–e). Roots exhibited especially strong labeling after 7 days (Fig. 3e), whereas the hypocotyl and cotyledon displayed weaker labeling in comparison (Fig. 3c, d). Figure 3d illustrates the transition between weak and strong labeling at the hypocotyl-root junction. We observed that the fluorescein azide was only able to penetrate the epidermis and/or the edges of the tissue and therefore visualization was limited to the cell layers that become accessible. Cutting or peeling of tissue prior to the fluorescein labeling was required to view internal cell layers.

### Phospholipase C treatment eliminates the fluorescence signal

If the fluorescence signal represents choline phospholipids, then it should be removable by digestion with phospholipase C (PLC). The bacterial PLC enzyme hydrolyzes the choline head group to produce PA, requiring calcium ions for its activity. We tested the effect of incubation of the propargylcholine-labeled seedling material with a bacterially derived phospholipase C (PLC) prior to the click-chemistry reaction. The imaging results indicate that the fluorescent signal from the fluorescein labeling is sensitive to the phospholipase C treatment (Fig. 3f–i). Therefore, the observed fluorescence represents primarily choline phospholipids.

### Propargylcholine labels choline phospholipids in tissues from mature plants

Since seedlings were able to incorporate propargylcholine, we next tested whether mature plants are able to maintain incorporation of the choline analog during their lifespan. *Arabidopsis* plants were grown to the mature/reproductive phase in Magenta boxes containing media supplemented with 200 μM progargylcholine or non-supplemented control media (see “Methods”). We did not observe differences in the physiological appearance between plants grown on supplemented versus control conditions (Fig. 2e). Plant tissues from both treated and control plants were harvested, fixed, and the click-chemistry reaction was performed with fluorescein azide (see “Methods”). Following fixation, leaf tissues were either cut into small strips or epidermal peels were prepared to allow the fluorescein azide to penetrate into the cells. Stem tissues were also cut into smaller pieces prior to performing the click chemistry.

**Fig. 4:**
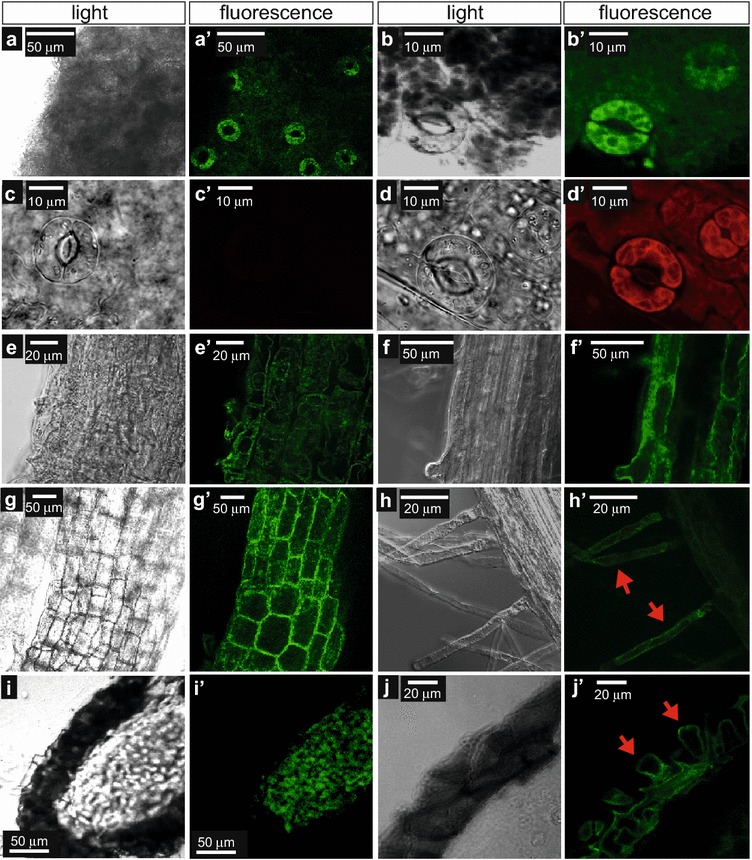
Visualization of labeled choline phospholipids in tissues from reproductive plants. *Arabidopsis* plants were grown for 6 weeks on agar media containing 200 µM propargylcholine. **a**–**j** Light and **a**′–**j**′ fluorescence matching light images are shown after performing click chemistry with fluorescein azide (green) (**a**, **b**, **e**–**j**) or Alexa Fluor 594 azide (red) (**c**, **d**). **a** Leaf epidermis reveals brightly fluorescing guard cells. **b** Magnification of guard cells on leaves. **c** The 0 μM propargylcholine control shows little or no background fluorescence (red) from the leaf epidermis after the Alexa Fluor 594 azide reaction. In contrast, **d** the epidermis from plants grown in on media containing propargylcholine display a strong fluorescence signal in guard cells, similar to that seen in **b**. **e**, **f** Root and **e**, **f** stem tissues exhibit fluorescence at cell peripheries and within cells. **h** Root hairs exhibit fluorescence (arrows). **i**, **j** Cryosections of mature seeds harvested at 7 weeks. **i** The seed endosperm exhibits a strong fluorescence signal. **j** Mucilage secretory cells of the seed coat exhibit fluorescence at the boundaries of the columella (arrows)

Similar to the control seedlings, the control plants grown without propargylcholine displayed little or no background fluorescence in the various plant tissues (Additional file 1: Figure S1). Mature plants grown on the supplemented media displayed visible incorporation of propargylcholine with labeling apparent in various tissues after 6 weeks of growth (Fig. 4 and Additional file 1: Figure S1). In leaves, labeling was detected most strongly in guard cells of the epidermis (Fig. 4a, b), suggesting that these specialized epidermal cells contain particularly high concentrations of choline phospholipids in comparison to the surrounding pavement cells. The guard cell labeling was also observed when the click chemistry reaction was performed with a different fluorescent label, Alexa Fluor 594 azide (Fig. 4c, d).

Labeling with fluorescein azide was readily detected in all cells from root and stem tissues (Fig. 4e–g), and was also visible in root hairs (Fig. 4h). Similar observations were made when the click-chemistry reaction was performed with Alexa Fluor 594 azide (Additional file 2: Fig. S2). Fluorescence was seen non-uniformly throughout cells in strands and patches in what appear to be intracellular membranes.

A subset of the plants was grown until they produced ripe seeds. Seeds from both treated and untreated plants were harvested, allowed to dry further for 1 week and sectioned using a cryostat (see “Methods”). Cryosections were treated with fluorescein azide. We observed labeling in the seed endosperm (Fig. 4i) and upon a higher magnification labeling was seen along the membranes of the columella in the mucilage secretory cells of the seed coat (Fig. 4j). Seeds collected from control plants grown in medium that lacked propargylcholine exhibited low levels of background fluorescence at the same microscope settings (Additional file 1: Fig. S1A).

### Characterization of choline phospholipid subcellular localization with dual labeling

**Fig. 5:**
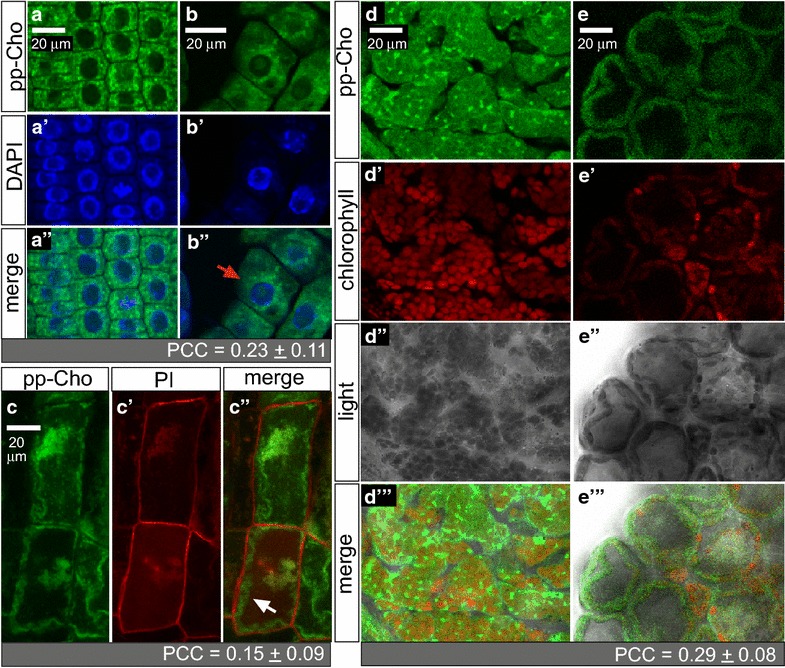
Dual visualization of choline phospholipids with nuclei, cell walls, and chloroplasts. *Arabidopsis* plants were grown for 6 weeks on agar media containing 200 µM propargylcholine (pp-Cho). **a**–**c** Roots were harvested and fixed, and fluorescein azide reactions were performed. Confocal laser scanning images show **a**, **b** root epidermal cells with fluorescein azide (green) marking propargyl-PC and **a**′, **b**′ DAPI fluorescence marking nuclei (blue). **a**″, **b**″ Merged images combine the fluorescence signals. Propargyl-PC signals typically surround (red arrow in **b**″) but do not overlay with nuclei. **c** Root epidermal cells were imaged after staining with propidium iodide (PI) to mark cell walls. **c** Fluorescein azide (green) indicates propargyl-PC and **c**′ PI marks cell walls. **c**″ Merged images show that the propargyl-PC occurs along cell walls (white arrow) but does not overlap with the cell wall stain. **d**, **e** Leaf samples were harvested and fluorescein azide reactions were performed followed by confocal microscopy. **d**, **e** Fluorescein azide (green) and **d**′, **e**′ chlorophyll fluorescence (red). **d**″, **e**″ Light microscopy. **d**‴, **e**‴ Merged images combine light microscopy overlaid with the fluorescence signals. Bright propargyl-PC foci appear independent of chlorophyll fluorescence. **d** Leaf mesodermal cells were imaged after epidermal layer was removed by peeling. A high density of chloroplasts (red) is visible. **e** Cross section showing fewer chloroplasts. Cell peripheries are marked by fluorescence signals from propargyl-PC. Co-localization was quantified using the Pearson correlation coefficient (PCC) (see Additional file 3: Table S1)

A series of fluorescent markers was used to assess the subcellular localization of the choline phospholipids signal. Co-localization was quantified using the Pearson correlation coefficient (PCC) (Additional file 3: Table S1). Double labeling with DAPI suggested that propargylcholine is only weakly associated with nuclei (Fig. 4a, b; PCC = 0.23 ± 0.11, Additional file 3: Table S1). However, labeling was observed to surround nuclei in dividing cells of the root, suggesting that this labeling may correspond to endoplasmic reticulum that contains choline phospholipids. In a similar manner, dual labeling with propidium iodide, a plant cell wall stain, revealed that the fluorescent signal was mostly excluded from cell walls (Fig. 5c, PCC = 0.15 ± 0.09, Additional file 3: Table S1). However, signal was apparent in regions adjacent to cell walls, possibly corresponding to the plasma membrane, endoplasmic reticulum and/or tonoplast.

To assess co-localization with chloroplasts we monitored chlorophyll autofluorescence. Observation of mesodermal leaf cells revealed bright fluorescent dots that weakly overlapped with chloroplast signals (Fig. 5d, e; PCC = 0.29 ± 0.08, Additional file 3: Table S1). Cells at the edge of the leaf exhibited very few chloroplasts, while labeling of propargylcholine was observed to occur strongly at cell peripheries (Fig. 5d).

**Fig. 6:**
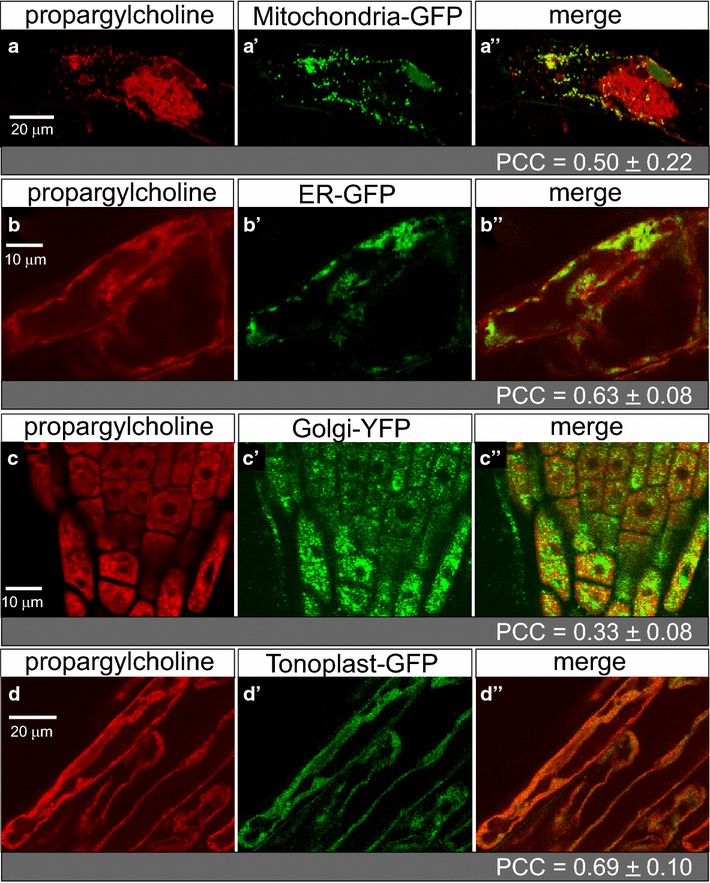
Dual visualization of choline phospholipids and GFP marker lines. *Arabidopsis* seeds were germinated and seedlings were harvested for click chemistry after 7 days on agar media containing 200 µM propargylcholine. **a**–**d** Alexa Fluor 594 azide (red) indicates propargyl-PC, **a**′–**d**′ GFP (or YFP) (green) marks a specified intracellular compartment, and **a**″–**d**″ merged images indicate overlapping expression as yellow or orange, as imaged by confocal laser scanning microscopy. **a** Hypocotyl epidermal cell shows localization of a mitochondrial marker in comparison to propargyl-PC; **b**–**d** root epidermal cells indicate localization of markers for **b** endoplasmic reticulum (ER), **c** Golgi apparatus, and **d** tonoplast. Co-localization was quantified using the Pearson correlation coefficient (PCC) (see Additional file 3: Table S1)

To further assess the intracellular membrane localization of the labeled choline phospholipids, click-chemistry experiments were performed with previously described GFP or YFP marker lines for the mitochondria, endoplasmic reticulum (ER), Golgi apparatus, and tonoplast [18]. To differentiate from the GFP (or YFP) signal, which overlaps with fluorescein azide (green), Alexa Fluor 594 azide (red) was utilized for the click-chemistry reaction with propargylcholine (Fig. 6). Mitochondria were visualized as dots in the cytoplasmic portion of cells that displayed moderate co-localization with propargylcholine labeling (Fig. 6a; PCC = 0.50 ± 0.22, Additional file 3: Table S1). Co-localization with mitochondria was variable, possibly reflecting the uneven distribution of these organelles within individual cells, relative to the propargylcholine signal (Fig. 6a). Considerable overlap of the propargylcholine fluorescence signal was observed with the ER (Fig. 6b; PCC = 0.63 ± 0.08, Additional file 3: Table S1). Co-localization with Golgi membranes was significantly weaker in comparison (Fig. 6c; PCC = 0.33 ± 0.08, Additional file 3: Table S1). In contrast, widespread overlap of the propargylcholine signal was seen with tonoplast membranes (Fig. 6d; PCC = 0.69 ± 0.10, Additional file 3: Table S1), which are cytoplasmic membranes that surround vacuoles, serving as an intracellular network for the transport of molecules to and from the vacuole and other associated organelles.

### Lipidomic analysis of propargylcholine incorporation

**Fig. 7:**
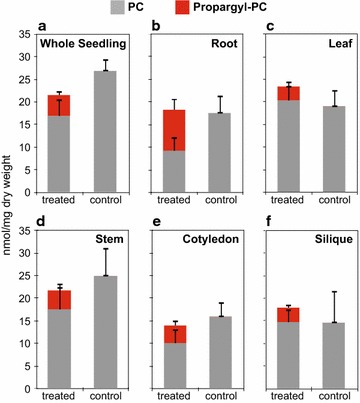
Quantification of propargyl-PC versus PC in plant tissues. Choline lipids were determined by ESI-MS/MS (see “Methods”; Additional files 4 and 5: Tables S2 and S3). PC (grey) and propargyl-PC (red) are represented in stacked graphs showing the replacement of PC by propargyl-PC in treated **a** whole seedlings, and **b**–**f** tissues from mature/reproductive plants: **b** roots, **c** leaves, **d** stems, **e** cotyledons, and **f** siliques. Treated seedlings and mature plants were grown in media supplemented with 250 and 200 µM propargylcholine, respectively. Controls were grown in media that lacked propargylcholine. Error bars indicate standard deviations for n = 5. No significant differences were found between the total PC + propargyl-PC versus PC in the treated and control samples, respectively (Two-tailed *t*-test, P < 0.005)

To determine how much propargylcholine was being incorporated into the various plant tissues and whether this incorporation affects the overall lipid composition of each tissue, electrospray ionization-tandem mass spectrometry (ESI-MS/MS) and lipidomic profiling was performed on whole seedlings and the mature plant tissues. Targeted scans were performed to detect intact propargyl-PC and propargyl-lysoPC in comparison to unlabeled phospholipids. The results indicate the strongest incorporation in the root with a total mole percent of propargylcholine amounting to ~50% of the total choline and propargylcholine lipids (Fig. 7; Additional files 4 and 5: Tables S2 and S3). Whole seedlings, leaves, stems, cotyledons, and siliques exhibited lower incorporation with ~22, ~13, ~19, ~28 and ~18% of total choline phospholipids, respectively. Besides propargyl-PC, propargyl-lysoPC was observed albeit at very low levels. In roots the incorporation of propargylcholine into lysoPC was the highest. The profiling data reveal that the incorporation of propargylcholine does not significantly impact the total amount of PC and PC-like lipid species in the various tissues. The total amount of PC and propargyl-PC in the treated samples is within the standard deviation of the total amount of PC in the untreated samples and a *t*-test (P < 0.005) showed no significant differences between the samples (Fig. 7).

**Fig. 8:**
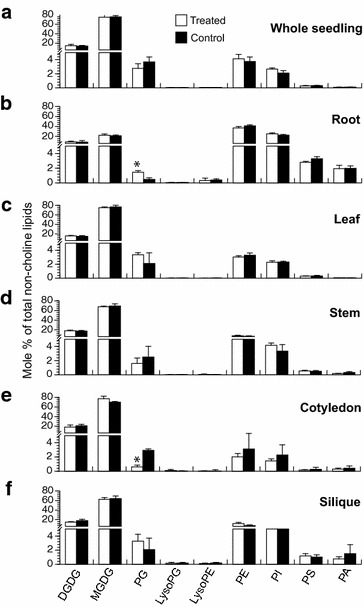
Profiles of non-choline lipids in plant tissues. Non-choline glycerolipids and phospholipids were determined by ESI-MS/MS (see “Methods”; Additional files 6 and 7: Tables S4 and S5). **a**–**f** The mole percent composition of total non-choline lipids is indicated for propargylcholine-treated (white) and untreated control (black) **a** whole seedlings and **b**–**f** tissues from mature/reproductive plants: **b** root, **c** leaf, **d** stem, **e** cotyledon, and **f** silique. The tissue samples are the same as those shown in Fig. 7. Error bars indicate standard deviations for n = 5. The treated and untreated samples have similar lipid profiles. However, in **d** the asterisk indicates a significant increase in PG in the untreated sample, and in **e** the asterisk indicates a significant decrease in PG (two-tailed *t*-test, P < 0.005)

Overall, incorporation of propargylcholine into propargyl-PC did not greatly impact the composition of non-choline lipids (Fig. 8; Additional files 6 and 7: Tables S4 and S5). However, quantification of the levels (nmol per gram dry weight) or the mole percent lipid of the non-choline lipids in the different tissues revealed a few differences between treated and control tissue. Notably, the amount of phosphoglycerol (PG) was significantly decreased in treated cotyledons at ~1.5 nmol per mg dry weight versus ~8.5 nmol per mg dry weight in untreated cotyledons, with a corresponding decrease in the mole percent PG (*t*-test, P < 0.005) (Fig. 8e; Additional files 6 and 7: Tables S4 and S5). Analysis of lipid composition in root samples indicated an opposite effect with a small but significant increase in PG (~0.41 nmol in the treated versus ~0.15 nmol per mg dry weight in the control) (*t*-test, P < 0.005) (Fig. 8b; Additional files 6 and 7: Tables S4 and S5). Correlating with lower levels of PG in cotyledons, we observed that during lipid extraction, the cotyledons from the propargylcholine-treated samples maintained their green pigmentation longer than the untreated controls for both the seedling and mature/reproductive plant samples. With exception of PG, all other lipid species that were analyzed including mono- and digalactosyldiacylglycerol (MGDG and DGDG), and the phospholipids lysoPG, lyso phosphatidylethanolamine (lysoPE), PE, phosphatidylinositol (PI), phosphatidylserine (PS) and PA, did not exhibit significant differences between the treated and control samples (*t*-test, P < 0.005) (Fig. 8; Additional files 6 and 7: Tables S4 and S5).

## Discussion

In the present work, we established a click-chemistry method using propargylcholine to specifically label choline phospholipids within plant cells and within various tissues of a plant. Unlike mass spectrometry imaging techniques that allow lipidomics *in situ* [19], this method can be performed without specialized equipment and software. Although we used *Arabidopsis* in our experiments, this approach may easily be applied to other plant species. We found that growing plants in media containing propargylcholine allows this choline bioortholog to be taken up by the roots into aerial plant tissues (including leaves, stems, cotyledons, siliques, and seeds) and incorporated into choline phospholipids within cell membranes. To visualize the incorporation in choline phospholipids, the plant material was fixed and a click-chemistry reaction was performed (Fig. 1). This copper ion catalyzed reaction, which was previously applied to mammalian cells [12], attaches fluorescein azide or another fluorescent probe such as Alexa Fluor 594 azide to the alkyne moiety on the incorporated propargylcholine.

The most efficient propargylcholine incorporation of ~50% was observed in roots (Fig. 7b), possibly due to the fact that the roots directly take up the bioortholog from the media. In 7-day-old seedlings, the root exhibits strong fluorescence labeling in comparison to the hypocotyl and cotyledon (Fig. 3c, d). It seems to take more time for the propargylcholine to be transported and incorporated in the aerial tissues. Lipidomic mass spectrometry data confirmed that intact propargylPC was present in all tissues analyzed, including cotyledons (Fig. 7). When plants were allowed to grow and mature in the presence of propargylcholine for a 6-week-period or longer, labeling was visible in all tissues prepared for microscopy, including seeds (Fig. 4i). Lipid profiling revealed that while the incorporation of intact propargylPC is less pronounced in the aerial tissues (Fig. 7), this degree of labeling was sufficient to visualize fluorescent signals (Fig. 4). For all tissues analyzed, the total propargyl-PC + PC in treated tissues was similar to that of total PC levels of the untreated controls. Furthermore, with the exception of abnormally low PG levels in cotyledons, and slightly higher PG levels in the root, the overall lipid profiles remained relatively unchanged (Fig. 8) and the propargylcholine labeled plants displayed normal physiology and development (Fig. 2). Taken together, these observations indicate that the propargylcholine is a bioortholog that can substitute for choline inside plant cells.

Similar to the case for in mammalian tissues as reported previously [12], we observed that plant tissues display propargylcholine incorporation into choline phospholipids in intracellular membranes (Figs. 4, 5, 6). In plant cells, the signals appeared to only weakly overlap with cell walls, and lined membrane regions adjacent to the cell wall (Fig. 5; Additional file 3: Table S1), consistent with biochemical studies that indicate high levels of PC in plasma membrane fractions from leaves [20]. We confirmed that the fluorescent label corresponds to choline phospholipids by incubating fluorescein-azide labeled roots with bacterial phospholipase C, an enzyme that hydrolyzes the choline head group. Detection of a fluorescent signal was sensitive to the enzyme treatment only in the presence of calcium, the co-factor that is needed for phospholipase C activity (Fig. 2f–i). Like in mammalian cells, we found the propargylcholine signal in plant cells to be only weakly associated with nuclei ([12]; Fig. 5a, b; Additional file 3: Table S1). In contrast, a moderate overlap was seen with mitochondria (Fig. 6a; Additional file 3: Table S1). Biochemical analysis of lipid composition of plant mitochondria has previously indicated that mitochondrial membranes are enriched in phospholipids and especially PC [21, 22]. The highest Pearson correlation coefficients were with a marker for the ER, a major site for phospholipid synthesis, and a marker for the tonoplast (Fig. 6; Additional file 3: Table S1). The co-localization with tonoplast is consistent with a previous report in which biochemical fractionation of membranes indicated very high levels of PC in the tonoplasts from etiolated pea seedlings [23].

We observed that the fluorescent choline phospholipid signal only weakly coincided with chloroplasts in leaf mesoderm cells (Fig. 5d, e; Additional file 3: Table S1). The thylakoid membranes are comprised mainly of the glycerolipids MGDG, DGDG, PG, and SQDG, and therefore strong co-localization with chloroplasts would not be expected [7, 24]. The lipid profiling data reported here reveal the abundant presence of these lipids in the photosynthetic leaf tissue with MGDG accounting for almost 80% of the total non-choline phospholipids (Fig. 8). Our results are consistent with a previous study in which the lipid contents of wild-type plant organs from *Arabidopsis* including leaf, stalk, flower, silique, root and seed tissues were quantified [25]. Similar to our results, the previous study also showed that glycerolipids including PG, MGDG and DGDG, are highly abundant in the leaf. Furthermore, these lipids (most notably MGDG) were also found to be abundant in stem, cotyledon and silique tissues. In contrast, the root contained lower levels of these lipids as is typical of a non-photosynthetic tissue.

While in general the labeling in the leaf was not observed to be as strong as in the root, the guard cells within the leaf epidermis showed exceptionally strong fluorescent labeling (Fig. 4a–d). This result was unexpected and it is not clear why guard cells appear to accumulate higher levels of choline phospholipids than the surrounding pavement cells of the epidermis. It is possible is that guard cells are more permeable to the click-chemistry reaction. If this is not the case, perhaps guard cells preferentially take up and accumulate the choline analog. Regulation of stomatal aperture by changes in turgor pressure may require a specialized lipid composition that is rich in choline-containing phospholipids. Lipid-based secondary messengers such a PA are implicated in stomatal closure functions [26, 27]. For example, PA, the product of Phospholipase D α1 (PLD α1), binds to and inhibits the protein phosphatase ABI1, resulting in abscisic acid (ABA)-induced stomatal closure [28]. More recently it was shown that in *pldα1* mutants, ABA-induced microtubule depolymerization and stomatal closure are impaired, signifying the importance of PA in this process [29]. In this regard, it is noteworthy that our observation of high levels of PC is associated with open guard cells under normal growth conditions.

## Conclusions

Our results show that choline phospholipids can be specifically visualized with a fluorescent probe when plants are grown in media containing the bioortholog propargylcholine. In addition to propargylcholine, azidocholine analogs have been reported to enable two-color imaging of choline phospholipids in double metabolic labeling experiments [30]. In our study, we demonstrate that propargylcholine labeling followed by click chemistry is a straightforward method to tag and visualize choline phospholipids within plant cells. This method represents a novel approach to imaging of lipids within different cell types, and in the present study has revealed intriguing differences among epidermal cells of the leaf. An analogous click-chemistry method is reported for a bioortholog of cholesterol in mammalian cells [31]. With the development of similar new bioorthologs, click-chemistry imaging of additional plant lipids, such as phytosterols, will be facilitated.

## Methods

A schematic overview of the steps involved in the described methodology is presented in Fig. 1: (1) Uptake and Incorporation: labeling plants with the bioortholog, propargylcholine, (2) fixation: harvesting and fixing of plant tissues, (3) click chemistry: performing the click-chemistry reaction, and (4) fluorescence microscopy: imaging the labeled choline phospholipids.

### Plant material and plant growth

The *Arabidopsis thaliana* accession used was Columbia (Col-0). The fluorescent marker lines for the endoplasmic reticulum (ER-gk, CS16251), Golgi (G-yk, CS16255), mitochondria (mt-gk, CS16263), and tonoplast (vac-gk, CS16257), were previously described [18] and obtained from the *Arabidopsis* Biological Resource Center. To prepare seedling material, seeds were sterilized for 5 min in 1 mL 1% bleach solution followed by 5 min in 1 mL 70% ethanol solution. After sterilization, seeds were washed 5 times with water and sown on 100 mm square petri dishes containing 50 mL (or 100 × 15 mm round petri dishes containing 20 mL) of 1× Murashige and Skoog medium [32] with 1% sucrose and 0.8% agar (plant tissue culture grade).

### Addition of propargylcholine to the media

The synthesis of propargylcholine was previously described [12]. To add propargylcholine to the media, 12.5 µL of 1 M propargylcholine and a volume of 87.5 µL of water (for ease of spreading) was added to the surface of the media of a square petri dish, giving a final concentration of 250 µM propargylcholine. Similarly, 5 µL of 1 M propargylcholine and 95 µL of water was added to a round petri dish. The propargylcholine solutions were spread onto petri dishes with glass beads and allowed to be absorbed into the medium for at least 1 h before sowing seeds. Petri dishes were placed at 4 °C for 2 days and then transferred to a 23 °C growth chamber under continuous light conditions. After 7 days, seedlings were harvested for imaging or lipid extraction.

To prepare material from mature/reproductive plants, seeds were treated as above except that 50 mL plant growth medium and seeds were placed in sterile Magenta plant culture boxes. To add propargylcholine to the media, 10 μL of 1 M propargylcholine and a volume of 90 μL of additional water (for ease of spreading) was added to the Magenta box and spread with glass beads to give a final concentration of 200 μM. After seeds were sown, boxes were closed and placed at 4 °C for 2 days. Plants were grown at 23 °C under continuous light conditions as above. Plants were grown to maturity for 6–7 weeks depending on the type of tissue being analyzed.

### Imaging of roots and measurement of root length

Seven-day-old seedlings were imaged using a stereo microscope (Leica M165 FC). Root lengths were measured using Image J (https://imagej.net/Welcome). Statistical analysis was performed using a two-tailed *t*-test to assess differences between the mean values from the control and the propargylcholine-grown roots.

### Phospholipase C treatment

Seedlings were grown on agar media containing 100 μM propargylcholine for 10 days, followed by fixation in 4% formaldehyde in 100 mM PBS while rotating overnight. After fixation, the seedlings were washed in TBS pH 7.5 (150 mM NaCl, 50 mM Tris). Seedlings were then placed in 1 ml of one of the following four solutions: TBS containing 10 mM EDTA and 1 mg/ml BSA with or without 1.1 units of bacterial phospholipase C or TBS containing 10 mM CaCl_2_ and 1 mg/ml BSA with or without 1.1 units of a bacterial phospholipase C (Type XIV, *Clostridium perfringens*: P4039, Sigma). Seedlings were incubated overnight at 37 °C, followed by 3 washes in TBS prior to fluorescein azide click-chemistry treatment as described below.

### Tissue fixation and fluorescein azide or Alexa Fluor 594 azide staining

Tissues were fixed in 100 mM phosphate-buffered saline (PBS) pH 7.2 with 4% formaldehyde at 4 °C while rotating overnight. Tissues were then washed 3 times with 100 mM PBS pH 7.2. Fixed samples were processed immediately or stored at 4 °C indefinitely prior to performing click-chemistry reactions. Fluorescein azide (5-azido-fluorescein) was synthesized from 5-amino-fluorescein as previously described [33]. Alexa Fluor 594 azide was purchased from Molecular Probes (A10270). The click-chemistry reaction solution consisted of 100 mM Tris-buffered saline (TBS), pH 8.5, 1 mM CuSO_4_, 20 μM fluorescein- or Alexa Fluor 594 azide, and 100 mM L-ascorbic acid. Five mL of this solution was made by adding 250 μL of 2 M TBS, pH 8.5, 5 μL of 1 M CuSO_4_, 2 μL of 50 mM fluorescein- or Alexa Fluor 594 azide, 3.74 mL of water and 1 mL of 0.5 M ascorbic acid. The ascorbic acid was added last. The tissue was placed in the room temperature solution rotating for 30 min. It was then washed 3 times with 100 mM TBS pH 8.5, followed by one wash with 0.5 M NaCl, and one more wash with TBS. It was stored at 4 °C in TBS until visualized by confocal laser scanning microscopy. For DAPI staining, tissue previously prepared with fluorescein was mounted in a 1 μg/mL DAPI in water for imaging. Propidium iodide samples were prepared by placing seedlings (already stained with fluorescein) in 10 µg/mL propidium iodide in water for 3 min. Seedlings were rinsed 3 times prior to imaging.

### Laser scanning confocal microscopy

Fixed and stained tissues were mounted in water prior to microscopy with a Zeiss LSM 5 PASCAL or with a Zeiss LSM 700 AxioObserver system. Fluorescein was excited at 488 nm and detected with a band pass filter of 505–530 nm. Alexa 594 was excited at 555 nm and detected with a 585 long pass filter. GFP was excited at 488 nm and detected with a band pass filter of 505–530 nm. All other microscope settings such as pinhole, laser power, detector gains remained the same for treated versus untreated samples. Autofluorescence of chloroplasts was excited at 488 nm detected with a long pass filter of 560 nm. For DAPI or propidium iodide imaging, tissue was viewed on a Zeiss LSM 510 Meta system with an inverted microscope using a multi-track configuration. DAPI was excited at 405 nm and detected with a band pass filter of 420–480 nm. Propidium iodide was excited at 545 nm and detected with a long pass filter of 585 nm. Seed material was sectioned before imaging by freezing at − 24 °C in Tissue Tek^®^ O.C.T. (optimum cutting temperature) embedding medium, followed by cutting 30 micron sections using a Leica CM30505 cryostat.

### Quantification of co-localization

Co-localization of the fluorescence signals from fluorescein azide or Alexa Fluor 594 azide marking propargylcholine (pp-Cho) and various organelle markers (DAPI for nuclei; GFP or YFP for mitochondria, ER, Golgi or tonoplast), cell wall stain propidium iodide and chlorophyll fluorescence were quantified for the two separate channels for four regions of interest (ROI) from each confocal image using JACoP (Just Another Co-localization Plugin) on the public domain ImageJ software [34]. Pearson correlation coefficient (PCC) values were calculated to estimate the degree of co-localization. PCC values range from + 1 to − 1, with + 1 representing complete positive correlation and − 1 representing negative correlation and 0 value representing no correlation.

### Lipid extraction

For lipid extractions from 7-day-old seedlings, five replicates were collected with each sample consisting of one whole seedling. The following tissues were harvested from treated and untreated mature/reproductive plants after 6 weeks of growth: roots, leaves, cotyledons, and stems. Siliques were harvested from separate plants after 7 weeks of growth. Each tissue sample was pooled from 3 to 4 individual plants, and 5 replicates of each treatment were collected. Immediately after harvest, tissues were placed in 3 mL of 75  °C isopropanol with 0.01% butylated hydroxytoluene (BHT) in 50 mL glass tubes and incubated for at least 15 min. 1.5 mL chloroform and 0.6 mL water were added. Tubes were shaken for 1 h after which the extracts were removed. Plant tissues were re-extracted at least 5 additional times for at least 30 min each (including one overnight extraction at − 80 °C) in chloroform:methanol (2:1) containing 0.01% BHT. Extractions were repeated until all of the plant tissues were white. The extracts were evaporated to dryness under nitrogen. The resulting lipid samples were dissolved in 1 mL of chloroform for mass spectrometry analysis described below.

### Mass spectrometry and lipid profiling

Electrospray ionization-tandem mass spectrometry (ESI-MS/MS) was performed as described in the supplemental data of [35], with propargyl-PC and propargyl-lysoPC targeted methods as previously performed [12]. All lipids were quantified using internal standards of the same lipid class, except that propargyl-PC was quantified using the PC internal standards and the propargyl-lysoPC was quantified using the lysoPC internal standards. Calculation of the nanomolar amounts reported in the lipidomic profiling data is based on the assumption that the intensities produced by equimolar amounts of lipid analytes and their internal standards are equal.

## Additional files

**Additional file 1: Figure S1.** Comparison of fluorescein azide signals from untreated controls with propargylcholine-treated samples.

**Additional file 2: Figure S2.** Comparison of Alexa Fluor 594 azide signals from untreated controls with propargylcholine-treated samples.

**Additional file 3: Table S1.** Quantification of co-localization of pp-Cho with subcellular markers using Pearson correlation coefficient.

**Additional file 4: Table S2.** Levels (nmol/mg dry weight) of choline phospholipids in propargylcholine-treated and untreated control plants.

**Additional file 5: Table S3.** Mole percent of choline phospholipids in propargylcholine-treated and untreated control plants.

**Additional file 6: Table S4.** Levels (nmol/mg dry weight) of non-choline lipids in propargylcholine-treated and untreated control plants.

**Additional file 7: Table S5.** Mole percent of non-choline lipids in propargylcholine-treated and untreated control plants.

## Abbreviations

BHT: butylated hydroxytoluene
CDP: cytidine diphosphate
DAPI: 4′-6-diamidino-2-phenylindole
DGDG: digalactosyl diacylglycerol
ESI-MS/MS: electrospray ionization tandem mass spectrometry
GFP: green fluorescent protein
MGDG: monogalactosyl diacylglycerol
PA: phosphatidic acid
PBS: phosphate buffered saline
PC: phosphatidylcholine
PCC: Pearson correlation coefficient
PE: phosphatidylethanolamine
PG: phosphatidylglycerol
ppCho: propargylcholine
ROI: regions of interest
TBS: tris buffered saline
YFP: yellow fluorescent protein
