# Supplementary material for: Bioorthogonal click chemistry for fluorescence imaging of choline phospholipids in plants
Source: Plant Methods. 2018 Apr 18;14:31. doi: 10.1186/s13007-018-0299-2 (PMC5905148; doi:10.1186/s13007-018-0299-2)
Supplement: Supplementary file 1 — Additional file 1: Figure S1. Comparison of fluorescein azide signals from untreated controls with propargylcholine-treated samples. [file 13007_2018_299_MOESM1_ESM.pdf]

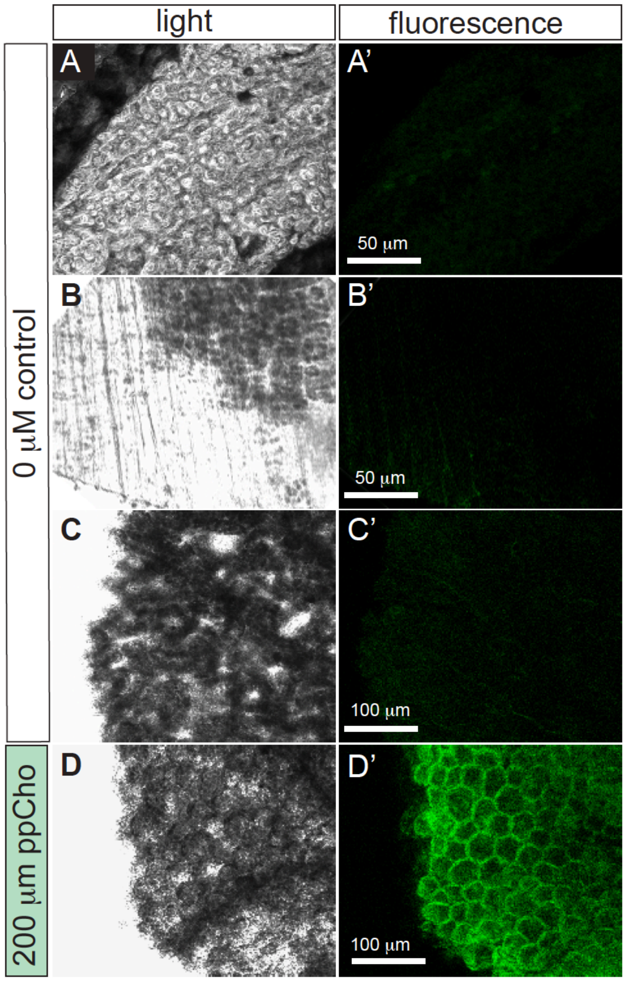

Additional file 1: Figure S1. Comparison of fluorescein azide signals from untreated controls with propargylcholine-treated samples. (**A-D**) Light microscopy show plant morphologies. (**A'-D'**) Fluorescence signals are indicated in green. Plants treated with **(A-C)** 0 μM propargylcholine exhibit little or no fluorescence. **(D)** Plants germinated and grown in 200 μM propargylcholine (ppCho) display strong fluorescence signals after fluorescein azide reaction followed by confocal laser scanning microscopy with settings held constant. **(A-C)** Samples from untreated plants: **(A)** Cryosection of mature seed showing endosperm, **(B)** epidermis of a stem, and **(C)** leaf in comparison to **(D)** propargylcholine-treated leaf tissue. This figure is supplementary to **Fig. 4**.
