# Supplementary material for: Bioorthogonal click chemistry for fluorescence imaging of choline phospholipids in plants
Source: Plant Methods. 2018 Apr 18;14:31. doi: 10.1186/s13007-018-0299-2 (PMC5905148; doi:10.1186/s13007-018-0299-2)
Supplement: Supplementary file 2 — Additional file 2: Figure S2. Comparison of Alexa Fluor 594 azide signals from untreated controls with propargylcholine-treated samples. [file 13007_2018_299_MOESM2_ESM.pdf]

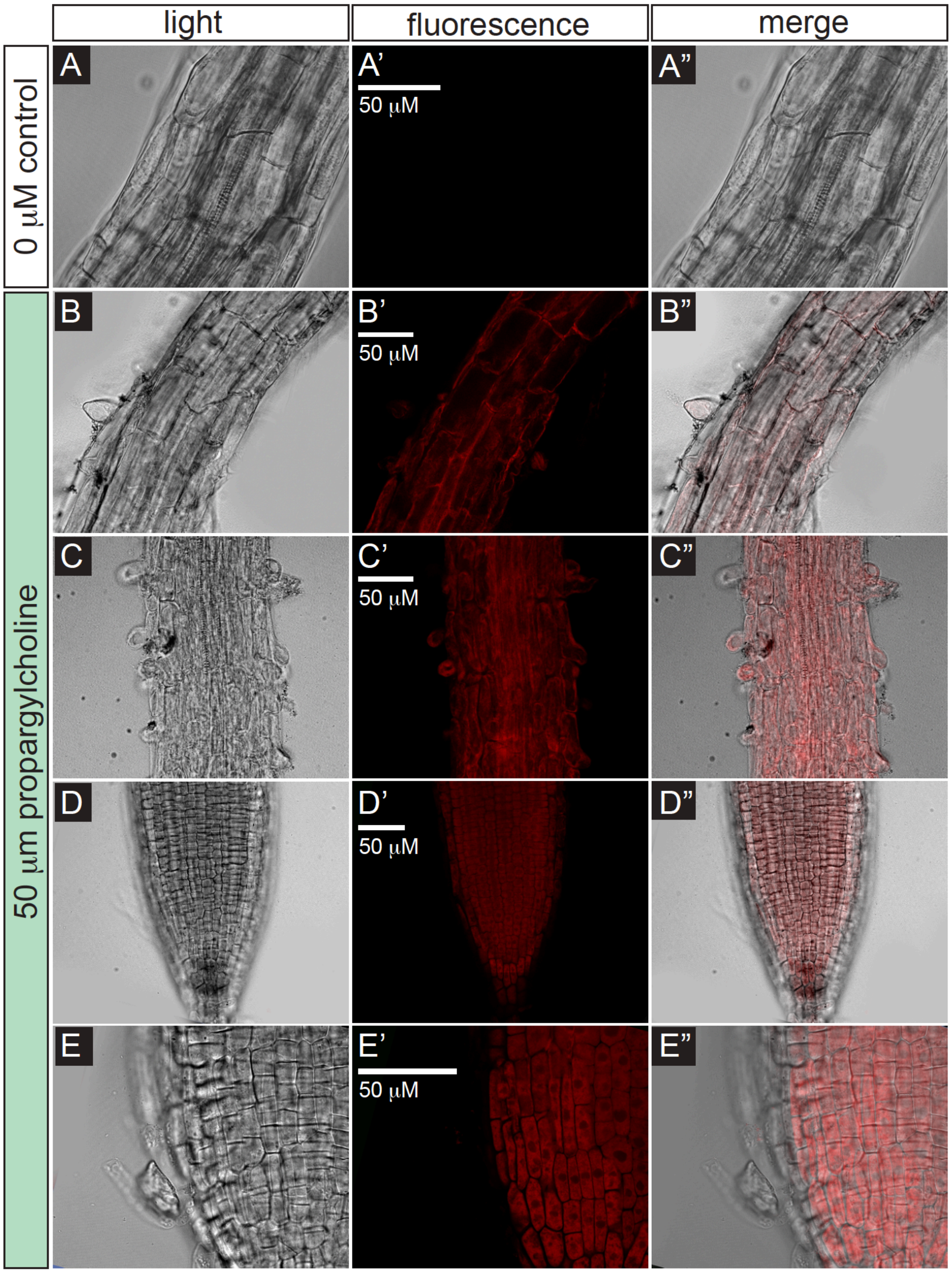

Additional file 2: Figure S2. Comparison of Alexa Fluor 594 azide signals from untreated control with propargylcholine-treated samples. (**A-E**) Light microscopy of plant tissues. (**A'-D'**) Fluorescence signals are indicated in red. (**A"-D"**) Merged images show overlay of light and fluorescence microscopy. Plants treated with **(A)** 0 μM propargylcholine exhibit little or no fluorescence, while **(B-E)** plants germinated and grown in 50 μM propargylcholine display a strong fluorescence signal after click chemistry with Alexa Fluor 594 azide, followed by confocal laser scanning microscopy with settings held constant. **(A-B)** Root epidermis, **(C)** root-hypocotyl junction, **(D)** root tip, and (**E**) root tip detail. This figure is supplementary to **Fig. 4**.
