# Supplementary material for: Bioorthogonal click chemistry for fluorescence imaging of choline phospholipids in plants
Source: Plant Methods. 2018 Apr 18;14:31. doi: 10.1186/s13007-018-0299-2 (PMC5905148; doi:10.1186/s13007-018-0299-2)
Supplement: Supplementary file 3 — Additional file 3: Table S1. Quantification of co-localization of pp-Cho with subcellular markers using Pearson correlation coefficient. [file 13007_2018_299_MOESM3_ESM.pdf]

Additional file 3: Table S1. Quantification of co-localization of fluorescence intensities with subcellular markers using Pearson correlation coefficient (PCC).

Cell type	Co-localization		Fig.	Pearson Correlation Coefficient (PCC)				
	**Channel 1**	**Channel 2**		**ROI 1**	**ROI 2**	**ROI 3**	**ROI 4**	**Mean (SD)**
Root epidermal	Fluorescein azide pp-Cho	DAPI - Nucleus	**4A**-1	0.26	0.09	0.27	0.25	0.22 (0.09)
			-2	0.40	0.06	0.10	0.32	0.22 (0.16)
			-3	0.13	0.37	0.32	0.20	0.25 (0.11)
								**0.23 (0.11)**
Root epidermal	Fluorescein azide pp-Cho	Propidium Iodide - Cell Wall	**4C**-1	0.10	0.21	0.17	0.29	0.19 (0.08)
			2	0.08	0.30	0.19	0.04	0.15 (0.02)
			3	0.03	0.17	0.04	0.17	0.10 (0.08)
								**0.15 (0.09)**
Leaf mesodermal	Fluorescein azide pp-Cho	Chlorophyll - Chloroplast	**4D**-1	0.17	0.15	0.18	0.28	0.20 (0.06)
			2	0.31	0.31	0.31	0.34	0.32 (0.02)
			3	0.42	0.28	0.30	0.41	0.35 (0.07)
								**0.29 (0.08)**
Hypocotyl epidermal	Fluorescein azide pp-Cho	GFP - Mitochondria	**5A**-1	0.16	0.32	0.73	0.29	0.37 (0.25)
			2	0.40	0.46	0.40	0.39	0.41 (0.04)
			3	0.82	0.72	0.55	0.80	0.72 (0.12)
								**0.50* (0.22)**
Root epidermal	Fluorescein azide pp-Cho	GFP - ER	**5B**-1	0.51	0.70	0.58	0.72	0.63 (0.10)
			2	0.58	0.67	0.49	0.73	0.62 (0.10)
			3	0.59	0.71	0.61	0.70	0.65 (0.06)
								**0.63**(0.08)**
Root epidermal	Alexa fluor 594 azide pp-Cho	YFP - Golgi	**5C**-1	0.32	0.34	0.27	0.28	0.30 (0.03)
			2	0.40	0.18	0.37	0.36	0.33 (0.10)
			3	0.25	0.29	0.41	0.50	0.36 (0.11)
								**0.33 (0.08)**
Root epidermal	Alexa fluor 594 azide pp-Cho	GFP - Tonoplast	**5D**-1	0.72	0.67	0.73	0.82	0.73 (0.06)
			2	0.72	0.78	0.78	0.79	0.77 (0.03)
			3	0.62	0.50	0.52	0.61	0.56 (0.06)
								**0.69**(0.10)**

Co-localization of the fluorescence signals from fluorescein azide or Alexa fluor 594 azide marking propargylcholine (pp-Cho) with various subcellular markers was estimated using the Pearson correlation coefficient (PCC). A PCC value of 0.0 indicates no association, while PCC values approaching 1.0 indicate a positive correlation. Four regions of interest (ROI) were examined from three confocal images for each marker. The first line in each row indicates PCC values for an image from **Fig. 5** or **6**. Means and standard deviations (SD) are also listed for two additional images. The total means and SD for n=12 ROIs are shown in bold. Significantly higher PCC values in comparison to the nuclear, cell wall, chloroplast, and Golgi markers were obtained for the mitochondrial marker (*Two-tailed *t*-test, $P < 0.03$), and for the ER and tonoplast markers (**Two-tailed *t*-test, $P < 10^{-7}$).
