# Supplementary material for: Bioorthogonal click chemistry for fluorescence imaging of choline phospholipids in plants
Source: Plant Methods. 2018 Apr 18;14:31. doi: 10.1186/s13007-018-0299-2 (PMC5905148; doi:10.1186/s13007-018-0299-2)
Supplement: Supplementary file 4 — Additional file 4: Table S2. Levels (nmol/mg dry weight) of choline phospholipids in propargylcholine-treated and untreated control plants. [file 13007_2018_299_MOESM4_ESM.pdf]

Additional file 4: Table S2. Levels (nmol/mg dry weight) of choline phospholipids in propargylcholine treated and untreated control plants.

Choline phospholipids	Treated seedling	Control seedling	Treated root	Control root	Treated leaf	Control leaf	Treated stem	Control stem	Treated cotyledon	Control cotyledon	Treated silique	Control silique
**PC**	16.913 (3.697)	26.856 (2.771)	9.201 (3.109)	17.533 (3.913)	20.323 (3.362)	18.981 (3.810)	17.553 (5.018)	24.901 (6.214)	10.110 (3.137)	15.910 (3.408)	14.735 (2.806)	14.560 (7.198)
**Propargyl PC**	4.644 (1.000)	0.013 (0.005)	9.205 (2.538)	0.026 (0.025)	3.103 (1.069)	0.030 (0.011)	4.188 (1.537)	0.048 (0.022)	3.887 (1.225)	0.050 (0.022)	3.252 (0.644)	0.047 (0.039)
**LysoPC**	0.039 (0.005)	0.064 (0.008)	0.075 (0.090)	0.151 (0.078)	0.033 (0.008)	0.126 (0.133)	0.135 (0.188)	0.103 (0.065)	0.164 (0.101)	0.091 (0.023)	0.112 (0.045)	0.241 (0.125)
**Propargyl LysoPC**	0.012 (0.002)	0.001 (0.001)	0.093 (0.115)	0.002 (0.002)	0.011 (0.005)	0.006 (0.002)	0.034 (0.046)	0.004 (0.003)	0.112 (0.041)	0.034 (0.025)	0.042 (0.011)	0.029 (0.037)

Lipid profiles were quantified from ESI-MS/MS data. Treated seedlings and mature plants (root, leaf, stem, cotyledon, and silique tissues) were grown in media containing 250 μM and 200 μM propargylcholine, respectively. Untreated control samples were grown without propargylcholine. Averages are shown for n=5 and standard deviations are indicated in parentheses. No significant differences were found between the total PC + propargyl-PC versus PC in the treated and control samples, respectively (Two-tailed *t*-test, $P < 0.005$). A graphical representation of the data for PC in comparison to propargyl-PC is given in **Fig. 7**.
