# Supplementary material for: Bioorthogonal click chemistry for fluorescence imaging of choline phospholipids in plants
Source: Plant Methods. 2018 Apr 18;14:31. doi: 10.1186/s13007-018-0299-2 (PMC5905148; doi:10.1186/s13007-018-0299-2)
Supplement: Supplementary file 6 — Additional file 6: Table S4. Levels (nmol/mg dry weight) of non-choline lipids in propargylcholine-treated and untreated control plants. [file 13007_2018_299_MOESM6_ESM.pdf]

Additional file 6: Table S4. Levels (nmol/mg dry weight) of non-choline lipids in propargylcholine treated and untreated control plants.

Lipid Analyzed	Treated seedling	Control seedling	Treated root	Control root	Treated leaf	Control leaf	Treated stem	Control stem	Treated cotyledon	Control cotyledon	Treated silique	Control silique
**DGDG**	33.205 (3.921)	46.070 (2.506)	2.557 (0.433)	2.439 (0.552)	58.559 (9.177)	41.318 (10.038)	27.306 (2.436)	38.843 (14.513)	46.782 (11.087)	59.840 (9.554)	13.067 (4.237)	15.231 (3.924)
**MGDG**	175.123 (58.219)	243.664 (35.744)	6.305 (1.247)	6.140 (0.720)	279.621 (20.723)	216.931 (60.356)	104.087 (13.323)	158.532 (65.224)	202.204 (46.000)	200.356 (36.057)	55.761 (18.655)	56.565 (23.178)
**PG**	6.223 (1.616)	11.695 (1.169)	0.413* (0.102)	0.145 (0.080)	12.494 (2.169)	5.663 (4.526)	2.569 (1.523)	5.096 (2.358)	1.541* (0.682)	8.504 (2.634)	2.757 (0.845)	1.854 (1.562)
**LysoPG**	0.072 (0.029)	0.101 (0.040)	0.012 (0.010)	0.019 (0.008)	0.064 (0.020)	0.080 (0.043)	0.033 (0.027)	0.065 (0.024)	0.286 (0.364)	0.116 (0.037)	0.113 (0.065)	0.135 (0.079)
**LysoPE**	0.136 (0.033)	0.192 (0.028)	0.091 (0.078)	0.112 (0.050)	0.060 (0.011)	0.077 (0.046)	0.098 (0.095)	0.087 (0.028)	0.097 (0.063)	0.084 (0.017)	0.086 (0.011)	0.154 (0.069)
**PE**	9.309 (1.712)	11.903 (1.017)	10.543 (2.154)	11.939 (1.972)	11.257 (1.561)	9.159 (2.114)	11.805 (3.345)	13.992 (4.105)	5.236 (1.442)	9.062 (2.519)	9.583 (1.551)	6.895 (4.159)
**PI**	6.088 (1.582)	6.723 (0.479)	7.127 (1.236)	6.559 (1.181)	8.484 (1.201)	6.495 (1.652)	6.393 (1.232)	7.073 (1.519)	3.713 (0.645)	6.612 (2.084)	5.071 (1.918)	4.706 (2.006)
**PS**	0.625 (0.171)	0.988 (0.227)	0.792 (0.144)	0.939 (0.062)	1.169 (0.240)	0.930 (0.262)	0.859 (0.277)	1.109 (0.224)	0.446 (0.176)	0.750 (0.176)	1.012 (0.299)	0.944 (0.647)
**PA**	0.179 (0.062)	0.286 (0.035)	0.540 (0.081)	0.575 (0.145)	0.252 (0.052)	0.169 (0.059)	0.300 (0.090)	0.746 (0.308)	0.748 (0.410)	1.177 (0.229)	0.631 (0.283)	1.171 (0.727)

Lipid profiles were quantified from ESI-MS/MS data. Treated seedlings and mature plants (root, leaf, stem, cotyledon, and silique tissues) were grown in media containing 250 μM and 200 μM propargylcholine, respectively. Untreated control samples were grown without propargylcholine. Averages are shown for n=5 and standard deviations are indicated in parentheses. A graphical representation of this data is given in **Fig. 8**. Asterisks indicate a significant difference between the treated and control samples (Two-tailed *t*-test, $P < 0.005$).
