# Supplementary material for: Bioorthogonal click chemistry for fluorescence imaging of choline phospholipids in plants
Source: Plant Methods. 2018 Apr 18;14:31. doi: 10.1186/s13007-018-0299-2 (PMC5905148; doi:10.1186/s13007-018-0299-2)
Supplement: Supplementary file 7 — Additional file 7: Table S5. Mole percent of non-choline lipids in propargylcholine-treated and untreated control plants. [file 13007_2018_299_MOESM7_ESM.pdf]

**Additional File 7: Table S5. Mole percent of non-choline lipids in propargylcholine-treated and untreated control plants.**

| Lipids Analyzed | Treated seedling | Control seedling | Treated root | Control root | Treated leaf | Control leaf | Treated stem | Control stem | Treated cotyledon | Control cotyledon | Treated silique | Control silique |
|---|---|---|---|---|---|---|---|---|---|---|---|---|
| **DGDG** | 14.936 (2.730) | 14.427 (1.254) | 8.976 (1.386) | 8.663 (2.962) | 15.669 (1.066) | 14.826 (1.482) | 17.916 (1.329) | 17.277 (1.335) | 18.238 (4.246) | 20.964 (0.788) | 14.758 (0.826) | 17.965 (2.665) |
| **MGDG** | 75.065 (3.787) | 75.530 (2.851) | 22.201 (2.674) | 21.345 (1.643) | 75.281 (1.549) | 77.019 (3.216) | 67.919 (1.319) | 69.594 (4.321) | 77.097 (5.226) | 69.976 (1.176) | 62.655 (3.540) | 64.186 (5.222) |
| **PG** | 2.763 (0.655) | 3.689 (0.692) | 1.455* (0.215) | 0.488 (0.210) | 3.339 (0.309) | 2.108 (1.524) | 1.601 (0.761) | 2.504 (1.502) | 0.599* (0.252) | 2.922 (0.375) | 3.265 (0.986) | 2.074 (1.632) |
| **LysoPG** | 0.032 (0.014) | 0.031 (0.011) | 0.045 (0.041) | 0.065 (0.029) | 0.017 (0.004) | 0.027 (0.010) | 0.022 (0.017) | 0.032 (0.018) | 0.109 (0.137) | 0.040 (0.006) | 0.147 (0.116) | 0.161 (0.109) |
| **LysoPE** | 0.060 (0.005) | 0.060 (0.006) | 0.335 (0.319) | 0.388 (0.181) | 0.016 (0.002) | 0.026 (0.011) | 0.064 (0.063) | 0.042 (0.022) | 0.037 (0.023) | 0.029 (0.002) | 0.109 (0.052) | 0.184 (0.094) |
| **PE** | 4.136 (0.615) | 3.753 (0.634) | 37.101 (2.894) | 41.191 (2.191) | 3.019 (0.194) | 3.287 (0.315) | 7.583 (1.180) | 6.362 (1.131) | 2.016 (0.478) | 3.128 (0.542) | 11.463 (2.747) | 7.503 (1.396) |
| **PI** | 2.652 (0.179) | 2.116 (0.330) | 25.147 (1.831) | 22.600 (1.452) | 2.278 (0.217) | 2.312 (0.117) | 4.151 (0.319) | 3.331 (0.933) | 1.447 (0.270) | 2.265 (0.389) | 5.654 (0.441) | 5.400 (1.512) |
| **PS** | 0.277 (0.067) | 0.306 (0.057) | 2.791 (0.151) | 3.279 (0.286) | 0.312 (0.040) | 0.334 (0.074) | 0.551 (0.108) | 0.514 (0.098) | 0.171 (0.062) | 0.260 (0.021) | 1.194 (0.320) | 1.025 (0.320) |
| **PA** | 0.079 (0.025) | 0.090 (0.014) | 1.949 (0.450) | 1.981 (0.325) | 0.068 (0.011) | 0.061 (0.018) | 0.194 (0.040) | 0.343 (0.128) | 0.286 (0.148) | 0.416 (0.076) | 0.753 (0.317) | 1.501 (1.279) |

Lipid profiles were calculated in mole percent from ESI-MS/MS data in **Additional File 6: Table S4**. Treated seedlings and mature plants (root, leaf, stem, cotyledon, and silique tissues) were grown in media containing 250 μM and 200 μM propargylcholine, respectively. Untreated control samples were grown without propargylcholine. Averages are shown for n=5 and standard deviations are indicated in parentheses. Asterisks indicate a significant difference between the treated and control samples (Two-tailed *t*-test, P < 0.005).
